# Experimental and Model Calculation Research on Shrinkage of Hybrid Fiber-Reinforced Recycled Aggregate Concrete

**DOI:** 10.3390/ma18051183

**Published:** 2025-03-06

**Authors:** Lijuan Zhang, Meng He, Xinzhe Li, Changbin Li, Jun Zhao, Hai-Cui Wang

**Affiliations:** 1 School of Mechanics and Safety Engineering, Zhengzhou University, Zhengzhou 450001, China; zhanglj0526@zzu.edu.cn (L.Z.); hm99618@163.com (M.H.); lxz12102022@163.com (X.L.); lcb5009@163.com (C.L.); 2 School of Civil Engineering and Transportation, North China University of Water Resources and Electric Power, Zhengzhou 450046, China; 3 Faculty of Construction and Environment, The Hong Kong Polytechnic University, Hong Kong 999077, China; wanghaic2009@163.com

**Keywords:** recycled concrete, shrinkage, steel fiber, polypropylene fiber, hybrid fiber

## Abstract

Recycled aggregate concrete (RAC), which is made by replacing all natural coarse and fine aggregates with recycled aggregate, plays a significant role in improving the recycling rate of construction materials, reducing carbon emissions from construction, and alleviating ecological degradation issues. However, due to its low strength and significant shrinkage and deformation problems, RAC has limited application. The effort of fiber type, fiber admixture, and fiber hybridization on autogenous shrinkage were studied to improve the structural safety of building materials and broaden the application of RAC. Test results indicate that the shrinkage of RAC decreases with an increase in fiber admixture, and steel fiber-reinforced RAC is more resistant to shrinkage deformation than polypropylene fiber-reinforced RAC. The shrinkage deformation of the hybrid fiber group is smaller than that of the single fiber group, and the inhibition of shrinkage deformation is most effective when the volume fraction of steel fiber is 0.5% and the polypropylene fiber content is 1.5 kg/m^3^. At 120 days, the PF15SF05 mixture showed a 65.3% reduction in shrinkage compared with ordinary RAC. By merging the shrinkage deformation characteristics of fiber-reinforced RAC and introducing the fiber influence coefficient, three theoretical calculation models for autogenous shrinkage applicable to single and hybrid fiber-reinforced RAC were established based on the experimental data.

## 1. Introduction

Recycled aggregate concrete (RAC) is a type of concrete made by replacing all natural coarse and fine aggregates with recycled aggregate (RA), which are obtained by processing waste concrete through a series of processes, such as crushing and particle screening. The application of RAC is significant for improving the recycling rate of building materials, reducing the carbon emissions of building materials, and alleviating current ecological degradation [1]. Compared to natural aggregate concrete (NAC), the use of RAC in concrete building structures can save 10–20% of the construction material costs [2,3]. Despite these advantages, RA is derived from waste concrete, which not only has a large amount of cement mortar attached to the surface of the aggregate but also contains many micro cracks inside the aggregate [4,5]. The presence of these materials increases the porosity and crushing index of RA, which can lead to brittle damage due to multi-phase composite materials and shrinkage cracking in the actual construction [6].

The study of shrinkage behavior in RAC shows that the water loss from RA mainly occurs at an early stage, with shrinkage developing rapidly in the early stages and more slowly in the later stages [7]. The effect of the coarse aggregate replacement rate on shrinkage was more significant than the effect of reduced strength due to increased coarse aggregate replacement [8]. The primary difference in shrinkage observed between recycled and normal concrete material was found at an early age. Lv et al. [9] designed a test on the autogenous shrinkage of RAC with different water–cement ratios. The test results showed that RAC, with 50% and 100% replacement rates, exhibited 26% and 48% higher shrinkage deformation, respectively, compared to NAC at 180 days.

To compensate for the inherent defects of RAC, fibers can be incorporated into RAC to improve its shrinkage performance [10] and increase the crack resistance [11], making it a new type of green building material that meets building safety requirements. Afroughsabet et al. [12] added 1% volume of steel fiber to RAC, and the reduction in water absorption and shrinkage of RAC reached 23% and 15%, respectively, at 28 days. Steel fibers effectively stopped crack propagation. Polypropylene fibers (PF) could effectively reduce shrinkage deformation, but their enhancement effect was not significant when the fly ash content was low [13]. Wu et al. [14] measured the early age and long term free shrinkage properties of PF-reinforced RAC. The test results showed that the addition of PF played an important role in controlling the amount of shrinkage deformation of the RAC. X-ray industrial computed tomography (ICT) also showed that adding a small volume of PF into the RAC did not significantly change the porosity but altered the distribution pattern of pore sizes. Zaid et al. [15] added 25% RCA and 3% steel fibers (SF) to concrete, producing a green composite material with proper strength and low drying shrinkage deformation. Several studies have shown that the shrinkage of concrete decreases with an increase in fiber content [16,17].

Hybrid fibers can better inhibit the development of concrete shrinkage and deformation by complementing each other’s advantages due to the different characteristics of the fibers themselves [18]. Hybrid PF were more effective in reducing shrinkage of AAS concrete than macro PF alone [19]. Hybrid PF volume fractions of 0.6% macro and 0.1% micro PF were found to be the most effective. Ma et al. [20] mixed cellulose fiber and steel fiber in high performance concrete and found that the autogenous shrinkage increased with an increase in SF dosage, while autogenous shrinkage decreased with an increase in cellulose fiber content. The best inhibition of shrinkage deformation was achieved when the cellulose fiber content was 0.9 kg/m^3^, resulting in an autogenous shrinkage reduction of about 33% compared with NAC.

By summarizing the results of previous research, it can be seen that the effect of hybrid fiber on reducing shrinkage deformation of RAC is obviously better than that of single-type fiber. However, there is no systematic study or report on the effect of hybrid fibers on the shrinkage performance of full RAC (coarse and fine aggregates of recycled concrete are all recycled aggregates). Existing concrete self-shrinkage prediction models are primarily based on the influence of aggregate, water–cement ratio, temperature, and humidity, but they do not account for the influence of fibers, and there are a lack of models for predicting the shrinkage deformation of fiber-reinforced RAC. This paper analyzes the effect of fiber types (SF, PF, and hybrid fibers) on the autogenous shrinkage and deformation of RAC from initial preparation to 120 days. By fitting and analyzing the experimental results and combining them with the existing calculation model, a self-shrinkage prediction model for fully hybrid fiber-reinforced RAC is established.

## 2. Experiment

### 2.1. Materials

An ordinary Portland cement P.O 42.5 was used throughout the study. The performance indexes are shown in Table 1, and the test method followed according to the Chinese standard [21]. Grade I fly ash from power plants is used as an admixture to partially replace cement. The performance indexes of fly ash were tested by Chinese Standard [22] and are shown in Table 2. The properties of RCA and RFA were tested according to “Recycled coarse aggregate for concrete [23]” and “Recycled fine aggregate for concrete an mortar [24]”, respectively. The fineness modulus of the recycled fine aggregate is 3.3, and its particle size distribution is presented in Table 3. The particle size distribution and basic performance indexes of RCA and RFA are shown in Table 4 and Table 5, respectively. The steel fiber used in the test is end-hooked steel fiber, with a radius of 0.75 mm and a length of 35 mm. The polypropylene fiber is high strength bundled monofilament fiber, with a length of 9 mm, and a diameter of 50 µm. The superplasticizer (SP) is a polycarboxylic acid-type high-efficiency water reducer with a 27% water reduction rate, and its performance meets the Chinese Standard [25].

### 2.2. Concrete Mixture Proportion

The RAC was supplemented with steel and polypropylene fiber in both single and hybrid forms. The main test parameters are the type and content of fiber. The volume of polypropylene fiber was small, and the external mixing method was directly used. The dosage of single polypropylene fiber was taken as 0.9 kg/m^3^, 1.2 kg/m^3^, and 1.5 kg/m^3^. However, due to the larger volume, steel fiber would have a greater adverse effect on the workability of recycled concrete if added externally. Therefore, the equal-volume aggregate incorporation method was adopted here. The volume ratio of single steel fiber was taken as 0.5%, 1.0%, and 1.5%. One set of specimens were made with the basic mix without fiber, six sets with single fiber, and nine sets with hybrid fiber. Steel fiber is used to replace an equal volume of part of the coarse and fine aggregates, while keeping the sand ratio (40%) unchanged. The specific calculation method is shown in Equations (1) and (2). Because the volume of polypropylene fiber is very small, it is directly added, and its influence on the concrete workability is ignored. The calculations are shown in the following equation:

(1) $$\begin{array}{c} \frac{\Delta S}{\rho_{s}}+\frac{\Delta G}{\rho_{G}}=\frac{sf}{\rho_{sf}} \end{array}$$

(2) $$\Delta S:\Delta G=S:G\left(0.40\right)$$

where S and G are the amounts of RFA and RCA in the base mix ratio; sf is the amount of steel fiber in the base mix ratio; ∆S and ∆G are the amounts of coarse and fine aggregates being replaced; and $\rho_{s}$, $\rho_{G}$, $\rho_{sf}$ are the densities of RFA, RCA, and steel fiber, respectively.

The water-to-binder ratio was taken as 0.35. A total of 16 groups of concrete were designed, and the mix proportions are shown in Table 6. The compressive strength values are listed for the subsequent model calculations.

### 2.3. Experiment and Methodology

According to GB/T50082-2009 [26], a shrinkage performance test was conducted on a 100 mm × 100 mm × 515 mm prism. Before pouring the concrete, lubricant was brushed inside the test mold, and two layers of PVC film were laid, with one layer of lubricant evenly applied to each layer of film. After pouring, a sealing film was placed over the test specimen to isolate it from external moisture exchange.

**Figure 1:**
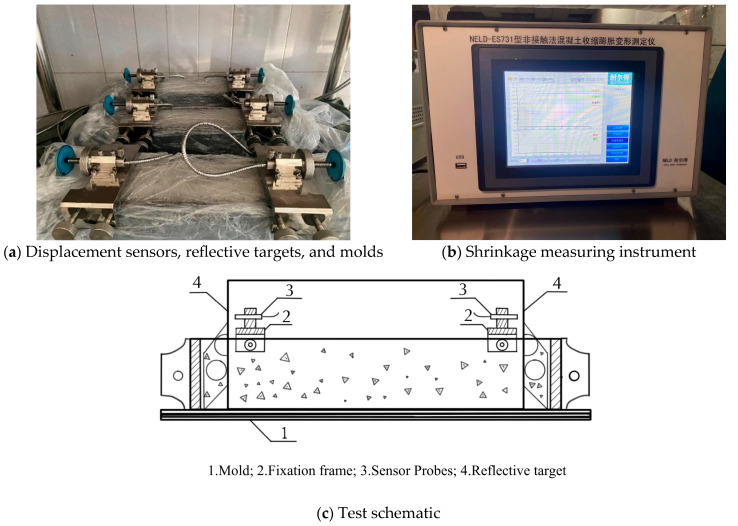
Shrinkage performance test equipment and schematic diagram.

As shown in Figure 1, a non-contact method was used for testing, and the data were recorded using a shrinkage expansion deformation tester with a high-performance embedded data acquisition system (model NELD-ES731), which was developed by Beijing NELD intelligent Technology Co., Ltd. (Beijing, China), the test data was recorded once every hour. The test environment was maintained at a temperature of (20 ± 2) °C and relative humidity of (60 ± 5)%. A digital temperature sensor was used to monitor the surrounding temperature and humidity. The target is locked firmly and kept vertical before the start of the test. No violent vibrations occurred during the test period, and it was strictly prohibited to touch the sensor, reflective target, or mold [26].

### 2.4. Experimental Data Processing

A non-contact concrete shrinkage tester is used to measure the autogenous shrinkage properties of concrete from pouring to 120 days of age. Test data were recorded every hour. The shrinkage values were calculated using Equation (3)

(3) $$\varepsilon_{st}=\frac{\left(L_{10}-L_{1t}\right)-\left(L_{20}-L_{2t}\right)}{L_{0}}$$

where $\varepsilon_{st}$ is the concrete shrinkage rate for the test period t (h); $L_{10}$ is the initial reading of the left sensor (mm); $L_{1t}$ is the reading of the left sensor at the test period t (h) (mm); $L_{20}$ is the initial reading of the right sensor (mm); $L_{2t}$ is the reading of right sensor test period t (h) (mm); and $L_{0}$ is the specimen measurement distance, which is equal to the length of the specimen minus the length of the two reflection targets buried in the specimen. In this paper, the specimen length is 450 mm. The calculated shrinkage values at different ages are shown in Table 7.

## 3. Test Results and Discussion

### 3.1. Influence of Polypropylene Fiber

**Figure 2:**
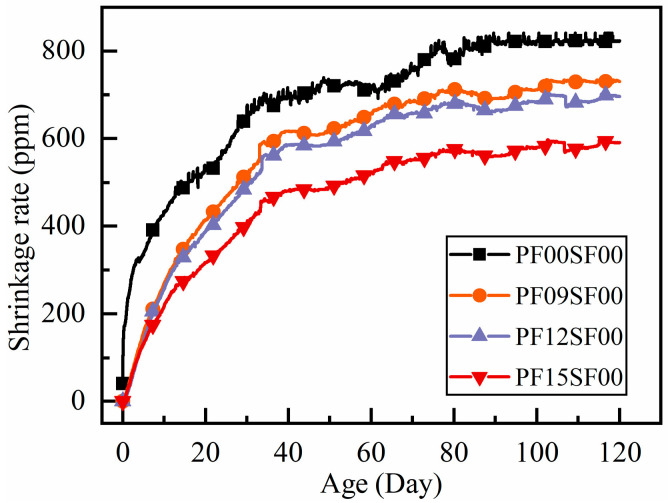
Shrinkage of RAC with age in different polypropylene fiber content.

In Figure 2, the shrinkage rate change curve of polypropylene fiber-reinforced RAC shows a similar trend to that of ordinary RAC, with a large increase in shrinkage rate in the early stages, followed by a gradual slowdown trend as the age increases. At 60 days of age, the shrinkage rate of polypropylene fiber-reinforced RAC did not change significantly, reaching about 90% of the 120-day value, while that of ordinary RAC reached 83.9% of the 120-day value at 60 days and continued to increase rapidly until the shrinkage rate stabilized after 80 days. Before 10 days of age, the shrinkage rate of all groups of polypropylene fiber-reinforced RAC exhibited changes during the early shrinkage stage. The differences in the shrinkage rates of the polypropylene fiber-reinforced RAC groups during the early shrinkage stage (before 10 days) were not significant, and all were notably lower than the shrinkage growth rate of ordinary RAC.

Calculation of shrinkage reduction in fiber concrete at various ages using Equation (4)

(4) $$\alpha =\frac{\lambda_{1}^{\alpha }-\lambda_{2}^{\alpha }}{\lambda_{1}^{\alpha }}$$

where α is the rate of shrinkage reduction; a is the age; *λ*_1_ is the shrinkage without fiber incorporation; and *λ*_2_ is the shrinkage with fiber incorporation.

**Figure 3:**
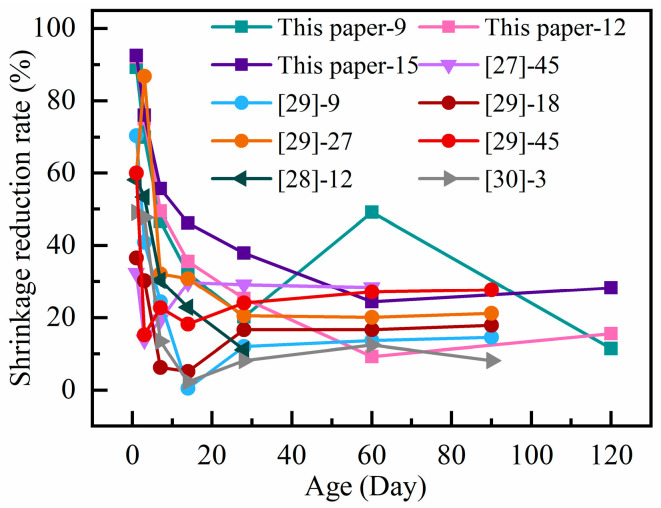
Relationship between shrinkage reduction rate and polypropylene fiber with age.

The test results from this work are compared with those from the previous literature in Figure 3 [27,28,29,30]. It illustrates how incorporating PF into RAC can significantly reduce the shrinkage of concrete. The shrinkage reduction follows a similar trend, gradually decreasing with age until it levels out. The reduction in shrinkage ranges from 8% to 28%.

Flexible PF, with its low modulus of elasticity, can effectively bond with mortar inside the matrix after being incorporated into RAC. This bonding improves the poor pore structure of RAC, reducing the number of capillary pores and internal porosity, ultimately lowering the total shrinkage rate [10,28]. The effects are as follows: the shrinkage change curve of PF-reinforced RAC stabilizes faster than that of ordinary RAC. The rate of shrinkage increases and the maximum shrinkage at the same age decreases with higher polypropylene fiber content. The improvement in shrinkage performance is particularly noticeable when the polypropylene fiber content reaches 1.5 kg/m^3^.

### 3.2. Influence of Steel Fiber

**Figure 4:**
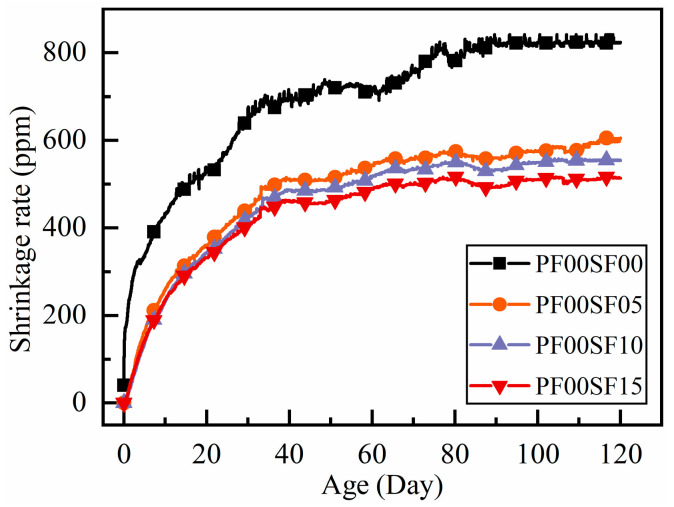
The shrinkage rate with age with different steel fiber dosage.

In Figure 4, the growth trend of the shrinkage for the four groups is similar, showing that the growth rate of shrinkage is large in the early stage, and then slows down when the age exceeds 30 days. The shrinkage rate of the three steel fiber-reinforced RAC specimens is very similar in the early stage but is far less than the shrinkage of the RAC without steel fibers. The maximum shrinkage rate at 28 days for SF05, SF10, and SF15 is only 68.3%, 64.8%, and 62.6% of that of the RAC, respectively.

**Figure 5:**
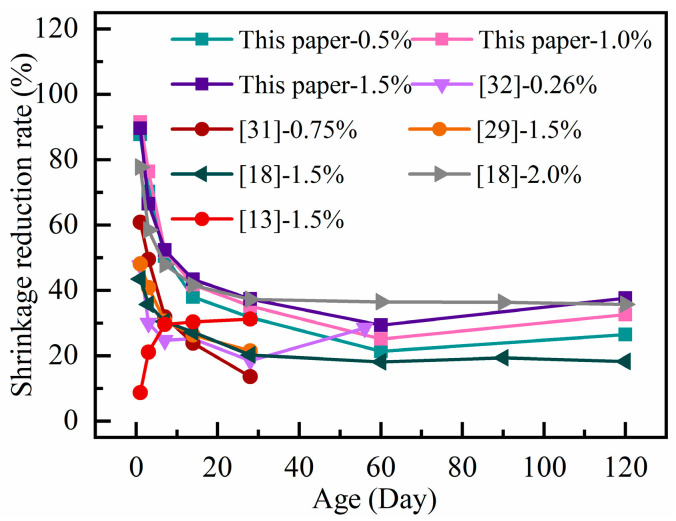
The shrinkage reduction rate with age with different steel fiber dosage.

The change rule of shrinkage reduction rate with age under different steel fiber dosages are shown in Figure 5. The experimental results in this paper are compared with those in the previous literature [13,18,29,31,32]. It can be seen that adding steel fibers significantly reduces shrinkage. The trend observed in the results of other studies is similar to the findings in this paper. Chen et al. [13] discovered that steel fibers played a crucial role in reducing concrete shrinkage, with the reduction stabilizing after 28 days, ranging from 17% to 35%.

From the above results, we can see that adding fibers can reduce the shrinkage of RAC, and the effects of steel fiber and polypropylene on the shrinkage of RAC are similar. The difference in shrinkage values is greatest within the first ten days, but the difference between the shrinkage rate of fiber-reinforced RAC and ordinary RAC gradually decreases as their age increases. At the same time, the difference between different fiber dosages gradually increases. This phenomenon may be due to both fibers improving the pore structure within the RAC during the early shrinkage stage. The fibers also reduce the water loss during the hydration reaction, which lowers the number of pores generated, thus improving the shrinkage performance of the RAC [18]. The contraction stress increases with age because the fibers are distributed throughout the concrete matrix. In the free shrinkage process, the uniform distribution of fibers forms a supportive network. A higher dosage of fibers can more effectively enhance the ability of RAC to resist shrinkage-induced tensile stress. The higher tensile strength of steel fiber plays a greater role in improving the tensile strength of the matrix, resulting in lower shrinkage for steel fiber-reinforced RAC compared to polypropylene fiber-reinforced RAC.

### 3.3. Influence of Hybrid Fiber

**Figure 6:**
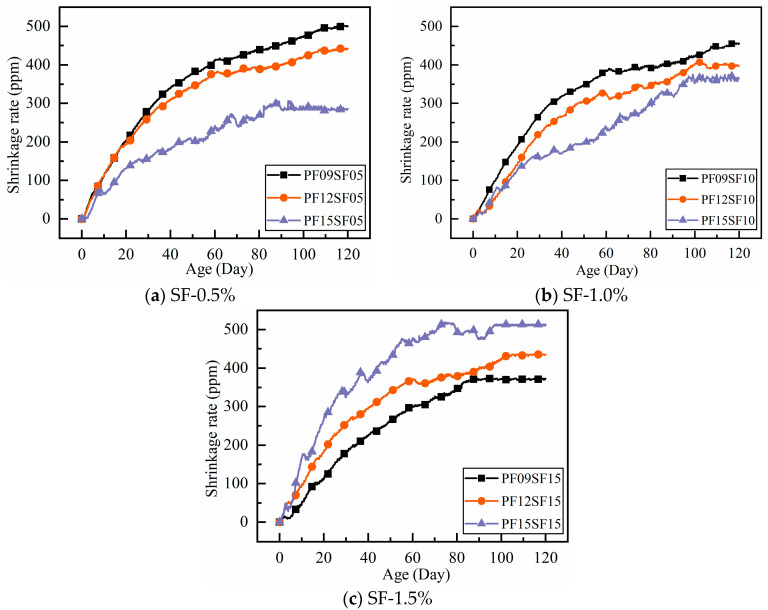
Shrinkage rate of RAC with different hybrid fiber content under different ages: (**a**) steel fiber volume ratio is 0.5%; (**b**) steel fiber volume ratio is 1%; (**c**) steel fiber volume ratio is 1.5%.

The test shrinkage data were plotted on a dashed line graph to compare and analyze the shrinkage performance of the hybrid fiber in RAC under different dosing conditions, as shown in Figure 6.

In general, the shrinkage deformation of RAC increased with age, with the fastest increase occurring in the first 14 days. After 40 days, the increase in shrinkage deformation began to level off, and after 60 days, the increase was minimal. The difference in shrinkage deformation compared to 120 days was negligible. The shrinkage rate of fiber-reinforced RAC was much smaller than that of regular RAC, and the shrinkage performance of steel fiber-reinforced RAC was better than that of polypropylene fiber-reinforced RAC. The shrinkage deformation of the hybrid group was smaller than that of the single fiber group, and the shrinkage rate of the PF15SF05 group at 120 days was the lowest.

In Figure 6c, the trend of shrinkage variation is significantly different from that of the hybrid group (SF-0.5%, 1.0%). The shrinkage variation in each group was significantly different before 10 days, and the difference gradually increased after 10 days. After 60 days, the shrinkage no longer changed significantly. It is worth noting that as the polypropylene fiber content increased, the overall shrinkage curve shifted upward, and the shrinkage value at each age increased. This trend was the opposite of the change pattern observed in the hybrid group (SF-0.5%, 1.0%).

The shrinkage of the hybrid group (SF-0.5%, 1.0%) gradually decreases with the increase in polypropylene fiber content, and the trend is exactly the opposite when the SF content is 1.5%. This may be due to the higher total volume fraction of fibers, which leads to the formation of voids between the cement paste and fibers, thus increasing the porosity of the concrete and accelerating water escape. The higher fiber volume reduces the concrete’s density, weakening the fiber bonding effect. For RAC materials with lower density and higher porosity, only the appropriate fiber content can more effectively improve shrinkage performance.

## 4. Shrinkage Calculation Model

The shrinkage calculation model is an empirical model based on the mechanisms of shrinkage and statistical analysis of a large amount of test data. It can predict shrinkage within a certain age more quickly. Accurately calculating shrinkage is crucial for researching and analyzing the shrinkage performance of RAC, and it is particularly important for the deformation control of concrete structures.

### 4.1. Comparison of Existing Calculation Model

**Figure 7:**
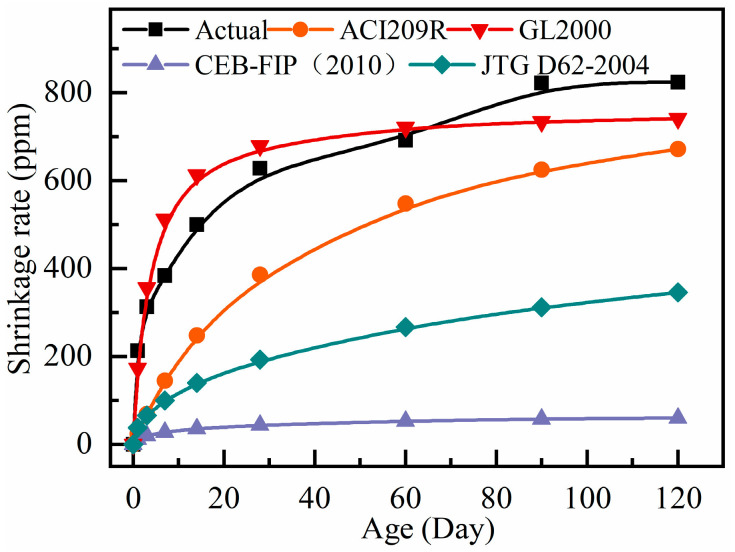
Comparison of measured values and calculated values of autogenous shrinkage of RAC.

Numerous studies on concrete shrinkage calculation models are currently being conducted both domestically and internationally. Since there are many variables that can affect shrinkage, various models use different sets of parameters. The theoretical shrinkage values at different ages were calculated using the equations from ACI PRC-209.2-08 [33], CEB-FIP (2010) [34], GL2000 [35], and JTG 3362-2018 [36]. The calculated shrinkage values were then compared with the test shrinkage values of ordinary RAC, as shown in Figure 7. To compare the differences between calculated and experimental values more intuitively, a difference coefficient λ was introduced. This coefficient compares the differences between the mean values of calculated and measured ratios for each group within 120 days of age. The equation for the difference coefficient λ is shown in Equation (5):

(5) $$\begin{array}{c} \lambda =|\frac{\sum_{i=1}^{120}\eta_{CA}}{120}-1|\;,\;\eta_{CA}=\frac{\varepsilon_{C}}{\varepsilon_{A}} \end{array}$$

where λ is the difference coefficient; $\varepsilon_{C}$ is the calculated value from the theoretical model; $\varepsilon_{A}$ is the measured value; and $\eta_{CA}$ is the ratio of the calculated value to the measured value at a certain age.

The test values of shrinkage strain and the difference coefficient λ are calculated from different specifications as listed in Table 8. The smaller the value of λ, the higher the accuracy of the theoretical model’s prediction.

From Figure 7, it can be observed that, except for the calculated values from the GL2000 prediction model, which closely follow the test value change curve, the calculated values from the other models are much smaller than the measured values. This is consistent with the calculation results of the difference coefficient λ, as shown in Table 8. The calculated values from CEB-FIP (2010) [34] and JTG 3362-2018 [36] are significantly smaller than the measured values of the autogenous shrinkage of common RAC, and the coefficients of variation λ are 0.931 and 0.697, respectively, indicating significant differences. The coefficient of variation λ for ACI209R is 0.476, which is closer to the measured values compared to CEB-FIP (2010) [34] and JTG 3362-2018 [36]. However, in the early shrinkage stage (before 10 days), the slope of the shrinkage rate change curve is small, and the growth rate of shrinkage is too slow, resulting in a larger deviation from the measured values. The λ value for GL2000 [35] is only 0.053, much smaller than the results of the other three models. Additionally, the growth rate is faster before 7 days, and the curve almost completely overlaps with the measured value. The curves are almost identical, and in the later stages of shrinkage development, they are also closer to the measured values than the other three models.

According to the aforementioned analysis, the GL2000 [35] model’s computed value and overall trend are more comparable to actual measured values than those from the models. In order to make the GL2000 [35] model more consistent with the actual shrinkage deformation, a correction factor is incorporated based on the model’s ability to forecast the RAC shrinkage values with hybrid fiber at various ages.

### 4.2. Modified Prediction Model

#### 4.2.1. Correction Based on GL2000 Model

The GL 2000 [35] prediction model values and the measured values of common RAC show good agreement in early stages of shrinkage. However, as age progresses, the two values begin to diverge, and the shrinkage rate at 120 days is underestimated. In this section, the accuracy of the GL 2000 [35] prediction model is improved by correcting the age-development function, while ensuring that the humidity function remains unchanged. The shrinkage strain correction coefficient and the relative humidity influence coefficient are fixed. The age–development function with the correction factor is shown in Equation (6):

(6) $$\begin{array}{c} \begin{array}{c} \beta \left(t\right)=\left[\frac{t-t_{c}}{at-t_{c}+0.15b\left(\frac{V}{S}\right)}\right] \end{array} \end{array}$$

where β(t) is the time–dependent shrinkage development factor; t is the calculated age; tc is the age at which shrinkage begins; V/S is the specimen body surface ratio in mm; and a, b are correction factors.

**Figure 8:**
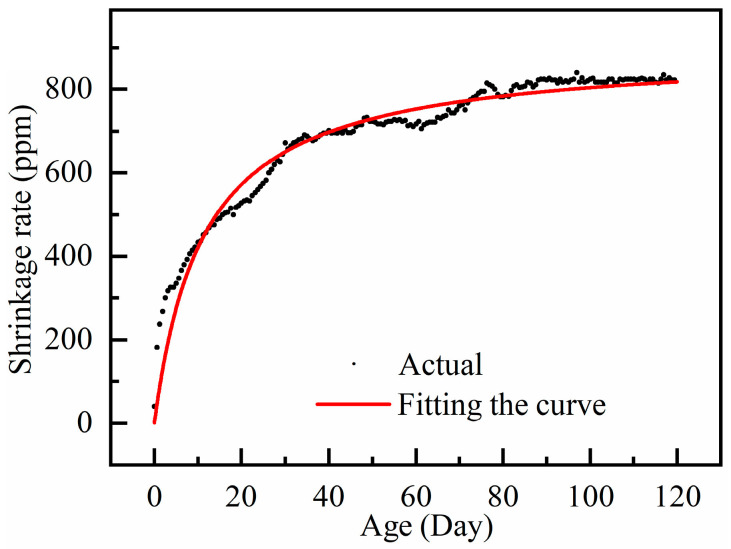
Correction curve of shrinkage rate using GL 2000 prediction model.

The measured data of RAC shrinkage were brought into the GL2000 model and Equation (6) was fitted to the actual shrinkage rate for analysis, as shown in Figure 8. The corrected coefficients, a and b, were obtained as 0.852 and 2.807, respectively, and the fitted correlation coefficient R^2^ was 0.979, indicating a strong correlation. The fitted prediction model is shown in Equation (7):

(7) $$\begin{array}{c} \varepsilon_{sh}=\varepsilon_{shu}\beta \left(RH\right)\left[\frac{t-t_{c}}{0.852t-t_{c}+2.807\times 0.15\left(\frac{V}{S}\right)}\right] \end{array}$$

where $\varepsilon_{sh}$ is the maximum shrinkage; $\varepsilon_{shu}$ is the shrinkage strain correction factor; and $\beta \left(RH\right)$ is the ambient relative humidity effect factor.

#### 4.2.2. Shrinkage Prediction Model Considering Fiber Impact

The autogenous shrinkage of fiber-reinforced RAC is significantly smaller than that of plain RAC. The experimental results show that fibers have a considerable influence on shrinkage deformation. Therefore, the RAC shrinkage prediction model (7) is modified by considering the effects of fiber content and type, and introducing the fiber influence coefficient ω. The modified fiber-reinforced RAC prediction model is shown in Equation (8):

(8) $$\begin{array}{c} \varepsilon_{sh,\omega }=\varepsilon_{shu}\beta \left(RH\right)\beta \left(t\right)\omega \end{array}$$

where $\varepsilon_{sh,\omega }$ is the predicted value of shrinkage, taking into account the effect of fibers; and ω is fiber influence coefficient.

**Figure 9:**
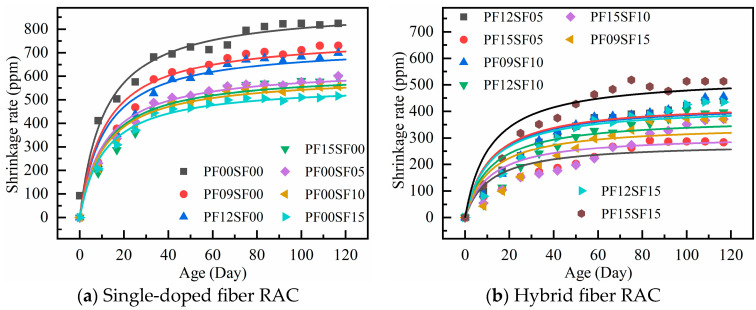
Comparison of shrinkage rate between measured values and GL 2000 modified model fitting curve with different fibers.

The measured data of 120-days shrinkage in 16 groups of specimens were substituted into Equation (8) and fitted to the actual shrinkage, as shown in Figure 9. The fiber influence factor ω and the fitted correlation coefficient R^2^ for each group were obtained, as shown in Table 9.

As seen from Figure 9 and Table 9, except for the PF12SF10, PF15SF10, and PF09SF15 groups, the fitted correlation coefficients for the other 16 groups are greater than 0.80, with the mean R2 value for all 16 groups being 0.8769, indicating a good fit. Since the shrinkage deformation of hybrid fiber-reinforced RAC is significantly different from that of single fiber-reinforced RAC, and the effect of the two fibers differ when mixed together compared to when used alone, the shrinkage deformation of fiber-reinforced RAC is predicted by classifying single fiber and hybrid fiber. This approach allows for a more accurate prediction of the shrinkage rate of fiber-reinforced RAC.

1. Single-mixed fiber RAC shrinkage prediction model

**Figure 10:**
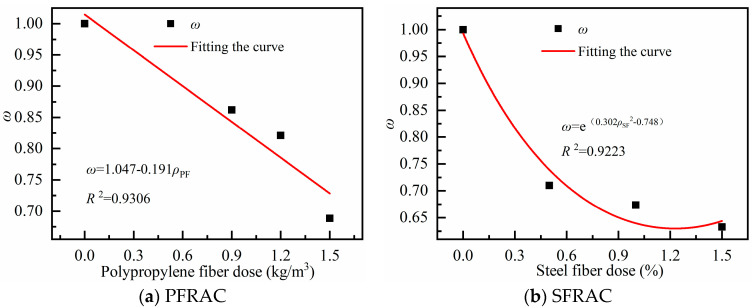
Relationship between fiber dose and fiber influence coefficient ω.

The shrinkage of RAC with different hybrid fibers of polypropylene and steel fibers were fitted with the fiber influence coefficients listed in Table 9. As shown in Figure 10, the equations for the fiber content and influence coefficients of the two fibers are obtained, as presented in Equations (9) and (10), respectively.

The PFRAC fiber dose and fiber influence coefficient ω are calculated as follows:

(9) $$\begin{array}{c} \omega =1.047-0.191\rho_{PF},\;R^{2}=0.9306 \end{array}$$

The SFRAC fiber dose and fiber influence coefficient ω are calculated as follows:

(10) $$\begin{array}{c} \omega =e^{\left(0.302\rho_{SF}{\;⠀}^{2}-0.748\right)},\;R^{2}=0.9223 \end{array}$$

where ω is the fiber influence coefficient, $\rho_{PF}$ is the polypropylene fiber dose (kg/m^3^); and $\rho_{SF}$ is the steel fiber dose, (%, by volume).

2. RAC shrinkage prediction model for hybrid fibers

The hybrid fiber characteristic coefficient β, can be calculated by Equation (11):

(11) $$\begin{array}{c} \beta =\varphi_{PF}\frac{l}{d}+\varphi_{SF}\frac{l}{d} \end{array}$$

where β is the hybrid fiber characteristic coefficient; $\varphi_{PF}$ and $\varphi_{SF}$ are the volume dosages of polypropylene fiber and steel fiber, respectively; and l/d is the fiber length-to-diameter ratio, where the steel fiber (l/d) ratio is 46.67 and the polypropylene fiber (l/d) ratio is 180. The characteristic coefficients for each group are shown in Table 10.

**Figure 11:**
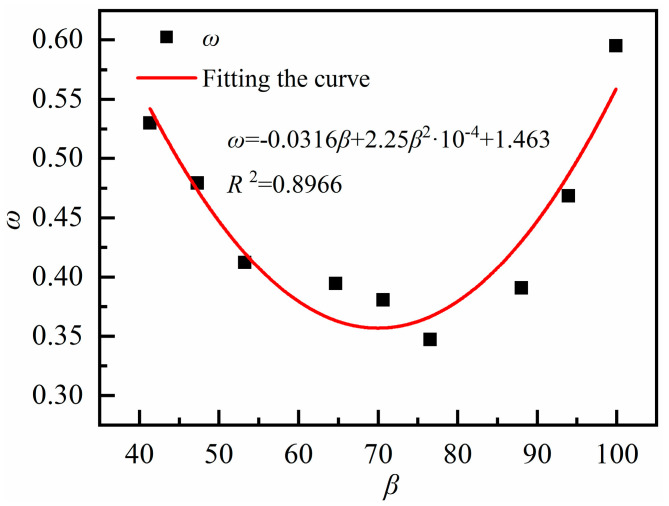
Relationship between characteristic coefficient β and fiber influence coefficient ω.

The characteristic coefficients of each group of blended fibers are fitted with the fiber influence coefficients in Table 9, as shown in Figure 11.

The relationship between ω and β is given by Equation (12).

(12) $$\begin{array}{c} \omega =-0.0316\beta +2.25\beta^{2}\times 10^{-4}+1.463,\;R^{2}=0.8966 \end{array}$$

In summary, the modified GL2000 contraction prediction model is:

(13) $$\begin{array}{c} \varepsilon_{sh}=\varepsilon_{shu}\beta \left(\mathrm{RH}\right)\left[\frac{t-t_{c}}{0.852t-t_{c}+2.807\times 0.15\left(\frac{V}{S}\right)}\right]\times \omega \end{array}$$

where, PFRAC ω can be calculated by Equation (9); SFRAC ω can be calculated by Equation (10); and hybrid fibers RAC ω can be calculated by Equations (11) and (12).

3. Shrinkage prediction model validation

For a more intuitive comparison of the accuracy of the prediction model, $\rho_{PF}$, $\rho_{SF}$, and β are substituted into Equation (13) to derive the calculated values of the shrinkage prediction model. The ratio of the measured values to the calculated values of the prediction model are calculated, as shown in Table 11. The mean, mean squared error, and coefficient of variation in the ratio between the measured and calculated values are presented in Table 12.

Table 11 and Table 12 demonstrate that, in the early stage of shrinkage, the predicted values for a few groups were overestimated. However, after 7 days, all measured values approached the predicted values more closely. The coefficient of variation in the ratio ranged from 0.058 to 0.333, with four groups exceeding 0.2, and the rest around 0.1. The mean ratio of the measured values to the predicted values ranged from 0.928 to 1.199. The mean variance of the ratio exceeded 0.3 in three groups, with the maximum not exceeding 0.35, while the rest were around 0.1.

This shows that the calculated theoretical values are close to the measured values, proving that the shrinkage prediction model for hybrid fiber RAC, based on the modified GL2000 model, is accurate and has a better practical application effect. It can provide valuable reference for the structural design of hybrid fiber RAC.

However, due to the establishment of this shrinkage model based on the test data of this study, only the hybrid of steel fiber and polypropylene fiber is used, so the model has certain limitations. For other types and additives of fibers, the fiber influence coefficient needs to be further fitted before it can be used.

## 5. Conclusions

A shrinkage performance test of 16 groups of RACs with different mix ratios over a period of 120 days were compared. The effects of different fiber types and mixing contents on the autogenous shrinkage deformation performance were analyzed, and the measured results were fitted and analyzed based on the prediction model. The prediction model for the autogenous shrinkage of hybrid fiber-reinforced recycled concrete based on the GL2000 model was established in the paper. According to the model, the shrinkage rate of recycled concrete with single fiber and hybrid fiber could be predicted directly according to the parameters such as the dosage of steel fiber and polypropylene fiber and the fiber length-to-diameter ratio. The following conclusions were drawn:

- The shrinkage deformation of RAC increases with age, with the fastest rate of increase occurring during the first 14 days. After 40 days, the increase in shrinkage deformation gradually levels off, and after 60 days the increase becomes minimal. The shrinkage deformation value at 120 days is not significantly different from that at later ages.
- The shrinkage rate of the fiber-reinforced groups is much smaller than that of ordinary recycled concrete. When a single type of fiber is used, the shrinkage rate decreases with the increase in fiber content at different ages. The shrinkage performance of steel fiber-reinforced RAC is better than that of polypropylene fiber-reinforced RAC. At 120 days, PF09, PF12, and PF15 showed reductions of 11.4%, 15.5%, and 28.3%, respectively, compared to ordinary RAC. At 120 days, SF05, SF10, and SF15 showed reductions of 26.5%, 32.6%, and 37.6%, respectively, compared to ordinary RAC.
- The hybrid fiber group exhibits less shrinkage deformation compared to the single fiber group. The optimal reduction in shrinkage deformation occurs when the volume fraction of steel fiber is 0.5% and the polypropylene fiber content is 1.5 kg/m^3^. At 120 days, PF15SF05 showed a maximal shrinkage reduction of 52.8% compared to PF00SF05. The shrinkage progressively decreases with an increase in polypropylene fiber when the volume fraction of steel fiber in the hybrid group is between 0.5% and 1.0%, while the trend is exactly the opposite when the steel fiber content is 1.5%.
- Four shrinkage prediction models were compared with the measured values, among which the GL2000 shrinkage prediction model was the closest to the measured values. The autogenous shrinkage prediction model for hybrid fiber-reinforced RAC was established by modifying the GL2000 model and considering the effect of the fiber.
- After the computed theoretical values were verified, the findings demonstrated limited dispersion and good agreement with the experimental values. This indicates good practical applicability and can provide valuable reference for the structural design of hybrid fiber-reinforced RAC.

## Figures and Tables

**Table 1:** Performance index of cement.

Index	Units	Standard Values	Test Value	Index	Units	Standard Values	Test Value
Compressive strength (3 d)	MPa	≥17.0	23.5	Specific surface area	m^2^/kg	≥300	345
Flexural strength (3 d)	MPa	≥3.5	5.6	Initial setting time	Min	≥45	230
Compressive strength (28 d)	MPa	≥42.5	44.8	Final setting time	Min	≤600	280
Flexural strength (28 d)	MPa	≥6.5	8.5	Loss on ignition	%	≤5.00	2.81

**Table 2:** Performance index of fly ash.

Index	Fineness Modulus	Bulk Density (kg/m^3^)	Moisture Content/%	SO_3_/%	Water Demand Ratio
Standard values	≤12.00	—	≤1.0	≤3.0	≤95
Test result	5.692	1120	0.10	0.933	92

**Table 3:** Particle size distribution of fine aggregate.

Sieve Size	4.75 mm	2.36 mm	1.18 mm	0.6 mm	0.3 mm	0.15 mm
Standard values	10~0	35~5	65~35	85~71	95~80	100~85
Residual of sieve	2	35	54	71	81	92

**Table 4:** Particle size distribution of coarse aggregate particle gradation.

Sieve Size	2.36 mm	4.75 mm	9.5 mm	16 mm	19 mm	26.5 mm
Standard values	95~100	90~100	40~80	—	0~10	0
Accumulated screening rate (%)	98.42	95.57	69.14	49.31	9.875	0

**Table 5:** Performance index of coarse aggregate.

Category	Apparent Density (kg/m^3^)	Crushing Index/%	Water Absorption Rate/%	Mud Content/%
Standard values	>2450	≤20.0	≤8.0	≤5.0
Test result	2612	16.4	6.95	0.4

**Table 6:** Mixture proportions and compressive strength of Hybrid fiber RAC.

Specimen Number	Mixture Proportions (kg/m^3^)								Compressive Strength (MPa)
	W	OPC	FA	RFA	RCA	WRA	PF	SF	
PF00SF00	200	457	114	628	942	5.484	0	0	38.87
PF09SF00	200	457	114	628	942	5.484	0.9	0	46.76
PF12SF00	200	457	114	628	942	5.484	1.2	0	49.49
PF15SF00	200	457	114	628	942	5.484	1.5	0	48.91
PF00SF05	200	457	114	623	935	5.484	0	39	52.70
PF00SF10	200	457	114	618	927	5.484	0	78	54.50
PF00SF15	200	457	114	613	920	5.484	0	117	54.63
PF09SF05	200	457	114	623	935	5.484	0.9	39	52.01
PF12SF05	200	457	114	623	935	5.484	1.2	39	60.98
PF15SF05	200	457	114	623	935	5.484	1.5	39	59.78
PF09SF10	200	457	114	618	927	5.484	0.9	78	63.36
PF12SF10	200	457	114	618	927	5.484	1.2	78	59.47
PF15SF10	200	457	114	618	927	5.484	1.5	78	56.52
PF09SF15	200	457	114	613	920	5.484	0.9	117	51.05
PF12SF15	200	457	114	613	920	5.484	1.2	117	57.05
PF15SF15	200	457	114	613	920	5.484	1.5	117	56.34

Note: PF09, PF12, and PF15 indicate that polypropylene fiber is mixed into recycled concrete at 0.9 kg/m^3^, 1.2 kg/m^3^, 1.5 kg/m^3^, respectively. SF05, SF10, and SF15 indicate that steel fiber is mixed into recycled concrete at 0.5%, 1.0%, and 1.5% by volume, respectively. W is water; OPC is ordinary silicate cement; FA is fly ash; RFA is recycled fine aggregate; RCA is recycled coarse aggregate; WRA is water-reducing agent admixture; PF is polypropylene fiber; and SF is steel fiber.

**Table 7:** Maximum shrinkage rate of RAC at different ages.

Test Number		Maximum Shrinkage Rate (ppm)					
	1 d	3 d	7 d	14 d	28 d	60 d	120 d
PF00SF00	213	313	384	500	628	692	824
PF09SF00	23	94	205	339	501	658	730
PF12SF00	17	78	194	322	469	628	696
PF15SF00	16	75	170	269	390	523	591
PF00SF05	26	93	190	310	429	544	606
PF00SF10	18	74	184	291	407	518	555
PF00SF15	22	105	183	283	393	489	514
PF09SF05	8	80	84	151	268	411	500
PF12SF05	4	90	82	153	251	379	442
PF15SF05	6	10	62	90	151	243	286
PF09SF10	3	26	70	141	256	384	456
PF12SF10	13	23	31	89	209	324	397
PF15SF10	1	16	36	78	164	236	364
PF09SF15	10	11	30	87	170	301	373
PF12SF15	7	44	70	137	243	371	435
PF15SF15	6	37	88	171	348	476	513

**Table 8:** Measured values of autogenous shrinkage at different ages of ordinary RAC and calculated values from prediction model.

Age (d)	Maximum Shrinkage Rate (ppm)				
	Measured Value	ACI209R	CEB-FIP	GL2000	JTG D62-2004
1	213	24.12	12.30	172.51	38.41
3	313	68.55	19.86	356.25	66.26
7	384	144.70	27.87	512.10	100.41
14	500	248.06	35.74	612.59	140.08
28	628	385.87	44.29	679.247	192.96
60	692	548.33	53.42	721.08	267.26
90	822	625.10	57.66	734.27	312.35
120	824	672.15	60.25	741.05	345.54
Coefficient of variation λ	0	0.476	0.931	0.053	0.697

**Table 9:** Prediction model fitting parameter values and correlation coefficients.

Test Number	ω	*R* ^2^	Test Number	ω	*R* ^2^
PF00SF00	1	0.979	PF12SF05	0.479	0.851
PF09SF00	0.862	0.940	PF15SF05	0.412	0.807
PF12SF00	0.821	0.935	PF09SF10	0.394	0.835
PF15SF00	0.689	0.928	PF12SF10	0.381	0.782
PF00SF05	0.710	0.975	PF15SF10	0.348	0.750
PF00SF10	0.674	0.968	PF09SF15	0.391	0.790
PF00SF15	0.633	0.979	PF12SF15	0.469	0.826
PF09SF05	0.530	0.818	PF15SF15	0.595	0.817
*R*^2^ Average	0.877				

**Table 10:** Fiber characteristic coefficients β.

Test Number	PF09SF05	PF12SF05	PF15SF05	PF09SF10	PF12SF10	PF15SF10	PF09SF15	PF12SF15
β	41.335	47.275	53.215	64.670	70.610	76.550	88.005	93.945

**Table 11:** Ratio of measured values to prediction model calculated values.

Test Number	1 d	3 d	7 d	14 d	28 d	60 d	120 d
PF00SF00	1.128	1.044	0.956	0.988	0.979	0.985	0.942
PF09SF00	1.276	1.152	1.064	1.08	1.014	1.057	0.980
PF12SF00	1.110	1.973	1.145	1.159	1.012	1.035	0.961
PF15SF00	1.068	0.908	1.034	1.002	1.044	1.156	1.052
PF00SF05	1.032	1.033	0.921	0.816	0.859	0.846	0.989
PF00SF10	1.389	1.161	1.144	1.046	0.962	0.942	0.905
PF00SF15	1.097	1.173	0.85	0.836	1.016	1.018	1.086
PF09SF05	1.236	1.073	1.109	0.977	1.088	1.047	0.885
PF12SF05	1.108	1.987	1.074	1.029	1.100	0.99	0.873
PF15SF05	1.090	0.876	1.115	1.106	1.168	1.070	1.097
PF09SF10	1.165	1.109	1.066	1.067	0.898	0.846	0.846
PF12SF10	0.984	1.892	0.910	0.969	1.079	0.868	0.728
PF15SF10	1.147	1.263	1.053	1.104	1.010	1.021	0.814
PF09SF15	1.298	1.064	1.055	1.020	1.194	1.122	0.931
PF12SF15	1.204	1.053	1.152	1.037	1.060	1.029	0.903
PF15SF15	1.105	0.805	1.149	1.199	1.011	0.921	0.879

**Table 12:** Mean, mean squared error, and coefficient of variation.

Test Number	Mean	Mean Square Error	Coefficient of Variation
PF00SF00	1.003	0.059	0.059
PF09SF00	1.089	0.091	0.084
PF12SF00	1.199	0.323	0.269
PF15SF00	1.037	0.069	0.066
PF00SF05	0.928	0.084	0.091
PF00SF10	1.078	0.156	0.145
PF00SF15	1.010	0.117	0.116
PF09SF05	1.059	0.101	0.096
PF12SF05	1.165	0.343	0.295
PF15SF05	1.074	0.085	0.080
PF09SF10	0.999	0.123	0.123
PF12SF10	1.061	0.353	0.333
PF15SF10	1.058	0.128	0.121
PF09SF15	1.097	0.111	0.101
PF12SF15	1.06	0.089	0.084
PF15SF15	1.009	0.137	0.136

## Data Availability

The original contributions presented in this study are included in the article. Further inquiries can be directed to the corresponding author.

## References

[1] Al Shouny A., Issa U.H., Miky Y., Sharaky I.A. (2023). Evaluating and selecting the best sustainable concrete mixes based on recycled waste materials. Case Stud. Constr. Mater..

[2] Dacić A., Mester-Szabó E., Fenyvesi O., Szalay Z. (2024). Life cycle assessment of concrete incorporating all concrete recycling products. Case Stud. Constr. Mater..

[3] Prasad D., Singh B., Suman S.K. (2022). Utilization of recycled concrete aggregate in bituminous mixtures: A comprehensive review. Constr. Build. Mater..

[4] Ouyang K., Liu J., Liu S., Song B., Guo H., Li G., Shi C. (2023). Influence of pre-treatment methods for recycled concrete aggregate on the performance of recycled concrete: A review. Resour. Conserv. Recycl..

[5] Ouyang K., Shi C., Chu H., Guo H., Song B., Ding Y., Guan X., Zhu J., Zhang H., Wang Y. (2020). An overview on the efficiency of different pretreatment techniques for recycled concrete aggregate. J. Clean. Prod..

[6] Wang D., Lu C., Zhu Z., Zhang Z., Liu S., Ji Y., Xing Z. (2023). Mechanical performance of recycled aggregate concrete in green civil engineering: Review. Case Stud. Constr. Mater..

[7] Khatib J.M. (2005). Properties of concrete incorporating fine recycled aggregate. Cem. Concr. Res..

[8] Jian S., Wu B. (2021). Compressive behavior of compound concrete containing demolished concrete lumps and recycled aggregate concrete. Constr. Build. Mater..

[9] Lv Z., Liu C., Zhu C., Bai G., Qi H. (2019). Experimental Study on a Prediction Model of the Shrinkage and Creep of Recycled Aggregate Concrete. Appl. Sci..

[10] Ahmed W., Lim C.W. (2021). Production of sustainable and structural fiber reinforced recycled aggregate concrete with improved fracture properties: A review. J. Clean. Prod..

[11] Gao D., Zhang L., Nokken M. (2017). Mechanical behavior of recycled coarse aggregate concrete reinforced with steel fibers under direct shear. Cem. Concr. Compos..

[12] Afroughsabet V., Biolzi L., Ozbakkaloglu T. (2017). Influence of double hooked-end steel fibers and slag on mechanical and durability properties of high performance recycled aggregate concrete. Compos. Struct..

[13] Chen W., Xie Y., Li B., Li B., Wang J., Thom N. (2021). Role of aggregate and fibre in strength and drying shrinkage of alkali-activated slag mortar. Constr. Build. Mater..

[14] Wu X., Zhou J., Kang T., Wang F., Ding X., Wang S. (2019). Laboratory Investigation on the Shrinkage Cracking of Waste Fiber-Reinforced Recycled Aggregate Concrete. Materials.

[15] Zaid O., Martínez-García R., Abadel A.A., Fraile-Fernández F.J., Alshaikh I.M.H., Palencia-Coto C. (2022). To determine the performance of metakaolin-based fiber-reinforced geopolymer concrete with recycled aggregates. Arch. Civ. Mech. Eng..

[16] Aghaee K., Khayat K.H. (2023). Design and performance of fiber-reinforced shrinkage compensating eco-friendly concrete. Constr. Build. Mater..

[17] Dong W., Yuan W., Zhou X., Zhao X. (2019). Influence of specimen geometries and drying conditions on concrete cracking in restrained elliptical ring tests. Constr. Build. Mater..

[18] Fang C., Ali M., Xie T., Visintin P., Sheikh A.H. (2020). The influence of steel fibre properties on the shrinkage of ultra-high performance fibre reinforced concrete. Constr. Build. Mater..

[19] Hassan A., ElNemr A., Goebel L., Koenke C. (2024). Effect of hybrid polypropylene fibers on mechanical and shrinkage behavior of alkali-activated slag concrete. Constr. Build. Mater..

[20] Ma R., Guo L., Ye S., Sun W., Liu J. (2019). Influence of Hybrid Fiber Reinforcement on Mechanical Properties and Autogenous Shrinkage of an Ecological UHPFRCC. J. Mater. Civ. Eng..

[21] (2023). Common Portland Cement.

[22] (2017). Fly Ash Used for Cement and Concrete.

[23] (2010). Recycled Coarse Aggregate for Concrete.

[24] (2010). Recycled Fine Aggregate for Concrete an Mortar.

[25] (2008). Concrete Admixtures.

[26] (2009). Standard for Test Methods of Long-Term Performance and Durability of Ordinary Concrete.

[27] Matalkah F., Ababneh A., Aqel R. (2022). Effect of fiber type and content on the mechanical properties and shrinkage characteristics of alkali-activated kaolin. Struct. Concr. J. FIB.

[28] Ding D., Zhang L., Zhao J., Li C., Wang Z. (2022). Effects of air-entraining agent and polypropylene fiber on the mechanical properties, autogenous shrinkage, and fracture properties of fully recycled aggregate concrete. Front. Mater..

[29] Vafaei D., Ma X., Hassanli R., Duan J., Zhuge Y. (2022). Microstructural behaviour and shrinkage properties of high-strength fiber-reinforced seawater sea-sand concrete. Constr. Build. Mater..

[30] Statkauskas M., Grinys A., Vaičiukynienė D. (2022). Investigation of Concrete Shrinkage Reducing Additives. Materials.

[31] Roberti F., Cesari V.F., de Matos P.R., Pelisser F., Pilar R. (2021). High- and ultra-high-performance concrete produced with sulfate-resisting cement and steel microfiber: Autogenous shrinkage, fresh-state, mechanical properties and microstructure characterization. Constr. Build. Mater..

[32] Kim D., Kim S., Choi W. (2021). Characteristics of Restrained Drying Shrinkage on Arched Steel Fiber-Reinforced Concrete. Appl. Sci..

[33] (2008). Guide for Modeling and Calculating Shrinkage and Creep in Hardened Concrete.

[34] Pan Z., Zhang H., Zeng B., Wang Y. (2023). Statistical Evaluation of CEB-FIP 2010 Model for Concrete Creep and Shrinkage. Materials.

[35] Delsaute B., Torrenti J.M., Nedjar B., Staquet S., Bourchy A., Briffaut M. (2024). Modeling compressive basic creep of concrete at early age. Mech. Time-Depend. Mater..

[36] (2018). Specifications for Design of Highway Reinforced Concrete and Prestressed Concrete Bridged and Culverts.

